# Macular Edema Following Silicone Oil Tamponade for Retinal Detachment: A Literature Review

**DOI:** 10.7759/cureus.51233

**Published:** 2023-12-28

**Authors:** Evgenia P Kontou, Christina Karakosta, Konstantinos Kounas, Ioannis Iatropoulos, Ioannis Tsinopoulos, Vassilios Kozobolis, Panagiotis Stavrakas

**Affiliations:** 1 Department of Ophthalmology, General Hospital of Athens “Korgialeneio-Benakio” Hellenic Red Cross, Athens, GRC; 2 Department of Ophthalmology, School of Medicine, University of Patras, Patras, GRC; 3 Department of Ophthalmology, National and Kapodistrian University of Athens School of Medicine, Athens, GRC; 4 Second Department of Ophthalmology, Aristotle University of Thessaloniki, Thessaloniki, GRC

**Keywords:** macular edema, oct (optical coherence tomography), retinal detachment surgery, silicone oils, pars plana vitrectomy

## Abstract

Macular edema (ME) is a major cause of reduced vision following intraocular surgery. Although the pathophysiology of ME is not completely understood, inflammatory mediators play a key role. The incidence of ME following pars plana vitrectomy with silicone oil tamponade varies between 13% and 27%. ME usually resolves spontaneously following silicone oil removal, but treatment may be required for resistant cases. In this review, the mechanisms of ME formation after pars plana vitrectomy, its incidence, and its possible therapeutic approaches are discussed.

## Introduction and background

Silicone oils (SOs) are liquid intraocular tamponades used in vitreoretinal surgery [1]. The use of SOs is linked with complications, including alterations of retinal micro and macrostructure, but the exact pathophysiologic mechanisms are not completely understood [2]. However, the alterations are associated with impaired retinal function and vision loss [2]. The evolution in retinal imaging, that occurred with the introduction of optical coherence tomography-angiography (OCT-A), opened new horizons in deciphering those microstructural and microcirculation changes of the retina [3]. This review aims to provide an updated and comprehensive overview of the mechanisms of macular edema (ME) formation after pars plana vitrectomy (PPV) with SO tamponade, its incidence, and its possible therapeutic approaches.

## Review

Methodology

This review was conducted between July 2023 and September 2023. For this literature review, relevant scientific articles were selected from various databases (PubMed, Google Scholar, and Science Direct) using keywords including retina, macular edema, silicone oil, and vitrectomy. MeSH terms were used as well (silicone oil, pars plana vitrectomy, optical coherence tomography, optical coherence tomography angiography, retinopathy, macular edema). Prospective and retrospective clinical studies, as well as narrative reviews, were considered. We limited our search to studies that were relevant to our subject, written in the English language, and published within the last 20 years.

Retinal detachment

Retinal detachment (RD) is the loss of adherence between the neurosensory retina and the underlying retinal pigment epithelium (RPE) [4]. The fovea lacks blood vessels and its oxygen requirements are met through the choroid, which supplies the oxygen and nutrition for the photoreceptors [5]. RD may cause irreversible damage to the photoreceptors and has an impact on overall retinal function. RD that involves the macula may lead to permanent loss of vision [4]. The incidence of RD varies from 1 in 10,000 to up to 18 in 100,000 [6]. The three types of RD are rhegmatogenous, tractional, and exudative [4]. Rhegmatogenous RD (RRD), the most common type, is the result of a retinal tear [7]. The break or hole allows liquified vitreous to pass into the subretinal space, detaching the retina [7]. This type of RD can occur over hours to months, depending on the location of the tear [8]. Risk factors for RRD include lattice degeneration, pathologic myopia, trauma, and a history of RD in the fellow eye [7,8]. Tractional RD is caused by the contraction of proliferative membranes [9]. When the force of the membrane contraction is strong enough, it can separate the neurosensory retina from the underlying RPE, leading to RD [4,10]. Risk factors for tractional RD include proliferative diabetic retinopathy, retinal vein occlusion, retinal vasculitis, uveitis, trauma, and proliferative vitreoretinopathy (PVR) [10,11]. Exudative RD is the result of excessive subretinal fluid accumulation without retinal breaks or tractional forces and is caused by a disruption in the integrity of the blood-retinal barrier [10,12]. Exudative RD may be of inflammatory, idiopathic, infectious, surgical, neoplastic, vascular, or drug-induced etiology [12].

Vitrectomy

Vitrectomy is the principal technique for RD repair. When performing PPV for rhegmatogenous RD repair, drainage of the subretinal fluid is performed and any traction on retinal breaks is released [13,14]. Chorioretinal adhesion at the borders of the tear is created through cryotherapy or laser, which leads to a scar that prevents future access of intraocular fluid to the subretinal space [15]. To prevent re-detachment during the period that is needed for the creation of a permanent seal (earlier with laser photocoagulation than retinal cryopexy), intraocular tamponade is necessary [16]. For the intraocular tamponade, the most common materials used are air, gas (mainly sulfur hexafluoride (SF6), and perfluoropropane (C3F8)), or SO [17]. The Silicone Study was a prospective multicenter randomized clinical trial, which compared three different tamponades for the repair of RD with PVR [14,17,18]. The three tamponades were 1,000 cs SO, 20% SF6, and 14% C3F8 [16-18]. The Silicone Study reported significantly better anatomic and visual outcomes with SO versus SF6 at one year, but no significant differences in anatomic or visual outcomes between SO and C3F8 [17,18]. The main indications for SO tamponade include complex retinal detachments due to PVR, giant retinal tears, recurrent detachments, traumatic RDs, and some cases of tractional RD [19].

Silicone oil

Polydimethylsiloxane is the main component of SO. SOs are available in two categories based on their viscosity, namely, 1,000 and 5,000 centistokes (cs) [20]. The different SO viscosity may affect the level of inflammation and the potential for complications. Higher-viscosity SOs may cause increased inflammatory responses compared to lower-viscosity ones.

SO is chemically neutral, lighter than water, allows light to penetrate and reach the retina, and is permeable to oxygen [20]. Moreover, SO is heatproof, which makes sterilization possible [20,21]. It has high surface tension and a refractive index of 1.404, due to which the optics of a globe filled with SO change based on the phakic or aphakic status [20,22,23]. In an eye with a crystalline lens, the anterior surface of the SO in contact with the lens takes a concave leading to a hyperopic shift of up to +5.00 diopters, while in an aphakic eye, the opposite occurs, with the convex surface of SO leading to a myopic shift of up to -5.00 diopters [20].

The most frequent postoperative complications of SO tamponade are intraconjunctival oil inclusion cyst, band keratopathy, cataracts, mostly as posterior subcapsular opacity, ocular hypertension, and glaucoma [24]. SO might be toxic for the optic nerve and the retina, causing ME in the latter [24-26]. After SO removal, transient hypotony, sudden vision loss, and macular microstructure alteration have been described among others [24].

Macular edema

Definition

ME is defined as an accumulation of fluid in the outer plexiform layer and the inner nuclear layer as well as a swelling of the Müller cells of the retina [27,28]. ME is an extension of the retinal extra or sometimes intracellular space locally in the macula [27-29]. It may be cytotoxic in origin, in which edema occurs within the cells, or more frequently vasogenic, in which fluid accumulation occurs between the cells [30]. The perifoveal retinal capillaries leak, causing an increased retinal thickness [27,29]. A cystoid macular edema is a configuration with radially oriented, perifoveal cystic spaces [27]. ME is defined as chronic when it lasts for more than six months [24,29]. Clinically, ME is identified by intraretinal cysts of differing numbers and sizes [31]. Nowadays, with the use of OCT, the diagnosis of ME is easily made by detecting well-demarcated intraretinal hyporeflective cysts on the retina, which cause elevated macular thickness [24,32].

OCT software can measure macular thickness in μm and is useful for monitoring the progression of ME. OCT software may also measure macular thickness in μm and that way the progression of ME may be followed [24]. It should be noted that any intraocular surgery, or certain procedures, such as argon laser treatment or cryotherapy, can potentially lead to ME.

Physiology

The accumulation of extracellular intraretinal fluid and proteins is prevented by several factors (osmotic forces, hydrostatic forces, capillary permeability, and tissue compliance) [27,29]. As a result, the rate of capillary filtration equals the rate of fluid removal from the extracellular retinal tissue, keeping the interstitial spaces of the retina dry in normal conditions [27]. In the retina, the inner blood-retina barrier (BRB) is formed by tight junctions between the endothelial cells of the retinal capillaries, while the outer BRB is formed by the tight junctions between the RPE cells [33]. The RPE actively transports fluid from the retina to the choroid. Inflammation within the vascular wall plays a key role in the development of ME [27,29]. Several inflammatory mediators are present and interact in a complex chain of reactions [34]. Those mediators include angiotensin II, vascular endothelial growth factor (VEGF), prostaglandins, cytokines, chemokines, matrix metalloproteinases, and interleukins [27].

ME after SO tamponade is associated with inflammatory as well as mechanical insults. Previous clinical and experimental studies have shown that SO stimulates the inflammatory reaction, which is macrophage-driven and persists for up to one month after SO removal [35-37]. Moreover, SO may cause intraocular inflammation by blocking the potassium ion buffering function of Müller cells [35]. This may lead to intraretinal toxicity and neuronal damage [35]. This hypothesis is supported by the fact that ME resolves with SO removal, thus, with the removal of the inflammatory nidus [35].

On the other hand, mechanical insults are represented by tractional forces. Previous studies have reported that SO is characterized by varying adhesive interactions with different materials and surfaces [35,38]. When this adhesive interaction occurs between SO and the retinal surface, tractional forces are created which may cause ME in a similar way to that of vitreomacular traction syndrome [35,39]. Again this hypothesis is supported by the resolution of ME following SO removal, which may relieve those tractional forces [35,39].

In addition to this, internal limiting membrane or epiretinal membrane peeling during the primary operation or in conjunction with SO removal may have a notable impact on the subsequent outcomes and potential complications, including the occurrence or resolution of ME. The removal of these membranes may influence the retinal architecture, affecting the likelihood of postoperative complications such as ME and subsequent visual outcomes.

Incidence

The incidence of ME may be affected by the grade of PVR and, as mentioned above, by the SO viscosity. The incidence of ME after SO tamponade varies between studies and possibly depends on the interval between the PPV and the SO removal [40]. The incidence of ME is reported to be 13.6% to 27.5%, but other studies have reported a much higher incidence of up to 36% [38]. Bae et al. conducted a comparative analysis of macular microstructure by OCT before and after SO removal [41]. The study evaluated 46 eyes following vitrectomy with SO tamponade and reported a 19% incidence of ME [41]. From these eyes, eight of nine recovered from ME within six months after surgery, accompanied by postoperative visual improvement [41].

Another study by Kiss et al. reported that ME was present in seven of 39 eyes operated on for RD complicated by PVR (grade C3 or worse) [42]. Rashad et al. studied 51 eyes with complicated RD including PVR, proliferative diabetic retinopathy, recurrent RD, penetrating trauma, uveitis, giant retinal tears, and macular holes and reported a similar incidence (seven eyes out of 51) using OCT [43]. The authors highlighted that with OCT scans, significantly more pathological changes were identified compared to clinical fundus examination [43]. In contrast, Karahan et al. reported that three of 24 eyes with RRD that received SO tamponade had ME, but in all three eyes, ME resolved within one month after SO removal [44]. In those eyes, the mean best-corrected visual acuity (BCVA) was significantly improved three months after SO removal compared to baseline. In those studies, silicone oil was removed after three to nine months. The study of Lo et al. evaluated 12 eyes and revealed significant changes (increase or decrease) in macular thickness in two distinct patient groups during SO tamponade compared to post-SO removal [45]. However, resolution of macular alterations occurred without further intervention within nine to 12 months after SO removal and was associated with improved BCVA in both groups [45].

The study by Yang et al. demonstrated that 21 eyes of 58 eyes developed ME. Of those, 13 eyes underwent SO removal and 11 experienced resolution of ME with or without further intervention [38]. The researchers reported that the presence of posterior staphyloma is significantly associated with ME after PPV with SO tamponade [38]. Shaheen et al. retrospectively studied 19 SO-filled eyes with ME. The authors reported that ME resolved in 10 eyes, seven of which occurred after SO removal. They also highlighted that eyes with PVR had a higher rate of persistent ME [46]. Er et al. investigated 65 eyes, which were divided into three groups based on the duration of silicone tamponade: ≤3 months, 3-6 months, and ≥6 months. They showed that there was a significant increase in central foveal thickness after SO removal in eyes with a duration of silicone more than three months and that the prevalence of ME was also significantly higher in the eyes with silicone duration of six months or longer [47].

Studies included in this review that reported the incidence of ME after SO tamponade are presented in Table 1.

**Table 1:** Macular edema (ME) incidence after silicone oil (SO) tamponade.

Study	Year	Eyes included	ME incidence after SO tamponade
Kiss et al. [42]	2007	39	18%
Bae et al. [41]	2012	46	20%
Karahan et al. [44]	2014	24	14%
Rashad et al. [43]	2016	51	17%
Lo et al. [45]	2017	12	33%
Yang et al. [38]	2018	58	36%

Treatment

As demonstrated in the aforementioned studies, the majority of ME cases related to PPV with SO tamponade may resolve spontaneously without any additional macular surgery after SO removal, probably due to the resolution of both inflammatory and mechanical insults [44].

However, for resistant ME after SO removal further treatment may be required. These treatment options for ME aim to limit the presence of inflammatory components, which are released from the disruption of the BRB [48,49]. These mediators cause dilation of vessels, elevated permeability of the capillaries, leukocyte attraction, and finally ME [48,49]. Nonsteroidal anti-inflammatory drugs (NSAIDs) block cyclooxygenase-1 and cyclooxygenase-2 and, thus, prostaglandin [48,49]. As a result, the ocular tissue penetrance of topical NSAIDs is a treatment of choice when dealing with ME, and they are effective even for chronic postoperative cases [49-51].

Corticosteroids are also effective in minimizing inflammation and cellular proliferation, as they also block prostaglandin and leukotriene production, and as a result, they increase the tightness of the BRB [52]. Periocular and intravitreal injections seem to be more effective for ME treatment compared to systemic steroids [53]. However, experimental studies have reported a decrease in the vitreous half-life of different drugs after PPV, and intravitreal triamcinolone acetonide is more rapidly cleared in vitrectomized eyes [54,55,56] In addition, intravitreal triamcinolone injection in eyes filled with SO has been reported as an option for the treatment of ME [55].

It is worth mentioning that dexamethasone has a 25-fold higher anti-inflammatory potency than cortisol [56]. The intravitreal injection of dexamethasone implant is considered to be an option for ME treatment with similar results between non-vitrectomized and vitrectomized eyes in decreasing central retinal thickness and improving visual acuity [57]. The implant slowly releases dexamethasone, gradually degrading into water and carbon dioxide, up to a complete degradation at almost six months post-insertion [56].

A combination of topical ketorolac and steroids seems to be more effective than the use of single agents for acute ME [49,58]. Carbonic anhydrase inhibitors are widely used for regulating the distribution of carbonic anhydrase in RPE, stimulating fluid resorption from the retina to the choroid [49,59].

The role of anti-VEGF intravitreal injections for the treatment of post-operative ME has been investigated in the past, and although in a study bevacizumab seemed to be effective for cases of pseudophakic ME, its use should be reserved for eyes unresponsive to conventional treatment modalities [60].

To date, the lack of evidence for the treatment of acute ME does not allow the suggestion of any particular practice as an efficient option [49,59].

## Conclusions

ME is a major cause of reduced vision following PPV with SO tamponade. Although the formation of ME is not completely understood, inflammatory mediators seem to play a significant role. ME usually resolves spontaneously following SO removal, but treatment may be necessary in resistant cases. Therapeutic approaches are similar to those applied for postoperative ME.
